# A Case Report of Culture-Negative Necrotizing Fasciitis With Low Laboratory Risk Indicator for Necrotizing Fasciitis (LRINEC) Score: A Diagnostic Dilemma

**DOI:** 10.7759/cureus.37268

**Published:** 2023-04-07

**Authors:** Azeem Rathore, Daniel A Reich, Nadim Qadir, Charles Harrison

**Affiliations:** 1 Internal Medicine, University of Florida College of Medicine – Jacksonville, Jacksonville, USA; 2 Medicine, University of Florida College of Medicine, Gainesville, USA; 3 Internal Medicine, Edward Via College of Osteopathic Medicine, Auburn, USA

**Keywords:** chat gpt, prognostic markers, comprehensive physical exam, clinical scoring system, necrotizing fasciitis, lrinec score

## Abstract

The Laboratory Risk Indicator for Necrotizing Fasciitis (LRINEC) score is a diagnostic tool used to help clinicians identify necrotizing fasciitis (NF) in its early stages. This tool uses six laboratory values including the patient's white blood cell count, C-reactive protein level, serum sodium level, creatinine level, and hemoglobin level to help with risk stratification. Each of these laboratory values is assigned a point value and the total score is used to determine the likelihood that a patient has NF, with a score of 6 or higher considered to be strongly indicative. The LRINEC score has gained popularity in recent years, having been included in guidelines and society recommendations for the management of NF. However, some studies have challenged the validity of the LRINEC score. Prompt and accurate diagnosis of NF is imperative given the associated mortality rate, which can be as high as 30%-40%, especially if the diagnosis is delayed. We present a case of a patient with a delayed diagnosis of NF that was initially missed in the early stages in the setting of a low LRINEC, however, growing clinical suspicion eventually led to an accurate diagnosis and management.

## Introduction

Necrotizing fasciitis (NF) is a rare but serious bacterial infection that affects the tissue beneath the skin, including the fascia and muscle. Also known as a “flesh-eating disease,” NF is caused by a variety of bacterial pathogens, most commonly Group A Streptococcus, but also can be caused by other bacteria such as *Staphylococcus aureus*, *Klebsiella*, *Escherichia coli*, and *Clostridium perfringens* that destroys the tissue it infects [1]. Symptoms include severe pain, swelling, redness, and a fever, often accompanied by vomiting and diarrhea; if not treated quickly, NF can lead to sepsis and death [2]. Indeed, NF is a life-threatening soft tissue infection that often requires emergent surgical intervention for debridement.

Diagnosis of NF can be challenging for several reasons. Firstly, as previously mentioned the early symptoms are fairly non-specific - fever, pain, and swelling can be seen in many other conditions [2]. Secondly, the rapid progression of NF can make prompt diagnosis difficult as the process of tissue destruction can occur within hours of initial symptoms. Third, the presentation of NF is highly variable in terms of the underlying cause, the location of the infection, and a patient's baseline medical history [3]. Early and accurate diagnosis of NF is imperative given the associated mortality rate can be as high as 30%-40%, especially if the diagnosis is delayed [1,4]. Designed in 2004, the Laboratory Risk Indicator for Necrotizing Fasciitis (LRINEC) score was a diagnostic tool used to help clinicians to identify NF in its early stages [5]. The score is calculated by adding assigned values that are based on six laboratory tests, white blood cell count, C-reactive protein level, serum sodium level, creatinine level, and hemoglobin level, and stratifies patients into “low,” “medium,” or “high” risk of NF with a score of 6 or higher considered to be strongly indicative of NF.

In recent years, the LRINEC score has gained popularity having been included in guidelines and society recommendations for the management of NF, including the Infectious Diseases Society of America (IDSA) and the Surgical Infection Society (SIS) [6]. While several studies have evaluated the performance of the LRINEC score in different patient populations showing high specificity for the diagnosis of NF other studies have also challenged the validity of the LRINEC score [6,7]. Within this context, we present a case of a patient who upon initial assessment had a low LRINEC score, but through additional history and physical examination data gathering clinical suspicion for NF trumped the score.

## Case presentation

We present a case of a 47-year-old female with a past medical history of right-sided triple negative *BRCA1*-positive invasive ductal carcinoma that presented to the emergency department (ED) for worsening left-sided breast pain for the previous two weeks. The patient's cancer history includes receiving neoadjuvant chemotherapy one year ago, which was followed by a skin-sparing bilateral mastectomy with immediate reconstruction with tissue expanders and a negative right axillary sentinel lymph node biopsy. However, her post-operative course had been complicated as she seemingly did not tolerate the tissue expanders resulting in multiple ED visits for pain, nausea, and vomiting. The patient later developed a seroma in the left breast which required further augmentation with permanent saline implants that were placed two months prior to her current presentation. Additionally, she had been treated with multiple courses of antibiotics in the setting of negative wound cultures. However, even after the surgical revision, the patient has had multiple ED visits for similar pains with her most recent visit one week in which she was discharged with antibiotics for suspected breast cellulitis. She was scheduled to have her left breast saline implant removed later during the week, but her plastic surgeon recommended she come to the ED for further evaluation.

On this visit, she endorses intractable pain and multiple episodes of non-bloody, non-bilious emesis over the past few days. She has been unable to eat or drink anything during this time. She also endorsed continued left-sided breast pain with radiation to her left shoulder. Her initial vitals included a blood pressure of 172/94 mm Hg, heart rate of 117 bpm, afebrile, and oxygenating at 100% on ambient air. On physical examination, no erythema, skin breakdown, or drainage was associated with the left breast or left shoulder. Laboratory results were unremarkable other than a hemoglobin of 11.1 g/dL, which was consistent with known chronic anemia. The patient was admitted to the medicine service for dehydration and concern about cellulitis. She was started on empiric antibiotics of ceftriaxone and doxycycline. On initial assessment, the patient had an LRINEC score of 1 due to her anemia (Table 1).

**Table 1:** LRINEC score for patient

Variables	Ranges	Value	Patient’s Score
C-reactive protein (mg/L)	< 150	4.49	0
	≥ 150		
White blood cell count (x10,000/µL)	< 15	4.04	0
	15-25		
	> 25		
Hemoglobin (g/dL)	> 13.5	11.1	1
	11-13.5		
	< 11		
Sodium (mEq/L)	≥ 135	140	0
	< 135		
Creatinine (mg/dL)	≤ 1.6	0.75	0
	> 1.6		
Glucose (mg/dL)	≤ 180	116	0
	> 180		

**Figure 1:**
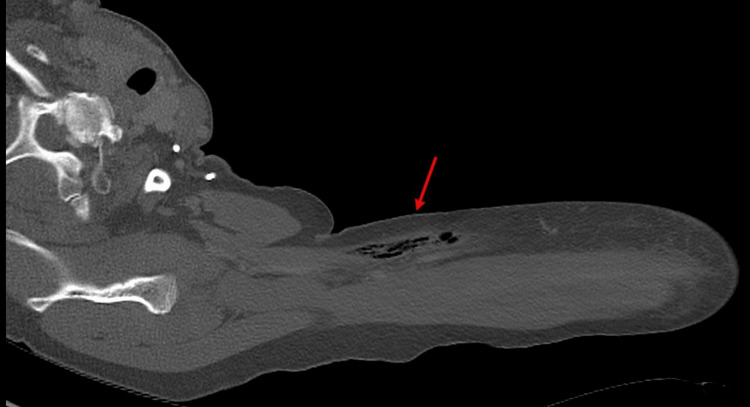
CT scan of left upper extremity showing multifocal regions of intramuscular air tracking along the left medial biceps muscle concerning NF.

Management centered on fluid rehydration and pain control. On day 2 of admission, however, the patient not only required six doses of morphine 4 mg but continued to endorse increased pain in her left upper extremity that appeared to be out of proportion to her unchanged physical examination including remaining afebrile. Her laboratory data continued to be unremarkable and consistent with an LRINEC score of 1 which was for a hemoglobin of 11.6 g/dL with the other parameters non-contributory. At this point in time, an immediate soft tissue ultrasound of the left upper extremity was ordered for increased suspicion of NF. In the interim, the patient was started on intravenous (IV) vancomycin. The soft tissue ultrasound findings were relayed by the on-call radiologist confirming the presence of subcutaneous air medial to the left biceps and an immediate upper extremity computed tomography was ordered that confirmed intramuscular air consistent within the tissue that was consistent with NF (Figure 1).

General surgery was subsequently consulted and within the hour they performed an urgent surgical debridement with resection that was without complication. Post-operatively, a vacuum-assisted closure was applied, and she was started on IV piperacillin/tazobactam, which was de-escalated to doxycycline and amoxicillin/clavulanic acid in the setting of both negative wound and blood cultures. The plastic surgeon was consulted, and he postponed the breast plant removal that was initially scheduled for later that week. On day 10 of admission, the patient was discharged with strict outpatient follow-up with general surgery and plastic surgery.

## Discussion

This case highlights the challenges of diagnosing NF and the limitations of the LRINEC score in predicting this condition. It also emphasizes the importance of maintaining a high index of suspicion for necrotizing soft tissue infections in patients with persistent pain that feels out of proportion to their presentation. Early recognition and prompt surgical intervention are crucial in achieving favorable outcomes for these patients. Indeed, NF is a clinical diagnosis, and the presence of intramuscular air on imaging studies, such as CT or MRI, can be highly suggestive of the condition, even in the absence of positive culture or histological findings - which was the case for our patient. In fact, up to 50% of cases of NF may have negative culture results, possibly due to prior antibiotic therapy, fastidious organisms, or inadequate sampling [8,9]. Again, as in our patient, she had received multiple doses of antibiotics beforehand which likely contributed to her confounding presentation. It was likely that if we had not incorporated the concerning physical examination findings into follow-up imaging modalities, the patient likely would have been discharged once her nausea and vomiting had subsided along with an opiate-based pain regimen. As described in the literature, most patients with NF have at least some abnormal laboratory values which intuitively makes sense since NF results from aggressive sepsis and should likely result in inflammation and leukocytosis [4,5]. However, our patient never had laboratory data to suggest sepsis prior to the imaging findings. It is possible that the NF had been developing for some time during even prior ED visits where no imaging was performed. Instead, following clinical suspicion led to prompt diagnosis and management of NF.

Indeed, over-reliance on diagnostic scoring systems, such as the LRINEC score, while helpful in risk-stratifying patients who are at high risk for NF, may lead clinicians astray from relying on clinical suspicion and gestalt [7]. For instance, in one study authors discussed the limitations and potential pitfalls of relying too heavily on clinical scoring systems such as the LRINEC score and emphasized the importance of incorporating clinical judgment and considering the overall clinical picture in making a diagnosis of NF [10]. In a 2017 editorial published in the *Journal of Hospital Medicine*, the authors argue that while clinical scoring systems like the LRINEC score can be helpful in diagnosing NF, they should not be relied on too heavily [11]. They note that these scoring systems are not foolproof and that other factors, such as clinical judgment and experience, should also be taken into account. There are several other case reports that discuss the pitfalls of LRINEC. For instance, a 2013 case reported a 37-year-old man that presented with NF with an LRINEC score of zero calling attention to clinicians to focus on clinical suspicion even if validated scores reveal no concern [12]. In another case, the authors described a patient who presented to a primary care clinic with symptoms of cellulitis but was ultimately diagnosed with NF after an initial misdiagnosis based on a low LRINEC score [13]. The converse has been shown to be possible as well: authors of a 2014 case report described a situation where reliance on the LRINEC score led to unnecessary diagnostic tests and delayed treatment in a patient with NF of the breast [14]. Finally, one retrospective study attempted to externally validate the LRINEC score but found that while the LRINEC score can be useful as a screening tool, relying too heavily on it lead in some cases to diagnostic delays and inappropriate treatment decisions [15].

Ultimately, the external validity of the LRINEC score may vary depending on the population being studied and the underlying prevalence of NF. Some studies have shown that the score has a high specificity and positive predictive value for identifying NF in populations with a high pretest probability of the disease, such as those with known soft tissue infections or those presenting with signs of sepsis [4]. However, the score may have lower sensitivity and negative predictive value in populations with a lower prevalence of NF or in cases where the disease is atypical or not fully developed [6]. As it stands, the LRINEC score has not been prospectively validated, and clinicians should remain aware of the limitations of this clinical decision tool. Therefore, the LRINEC score should be used in conjunction with clinical judgment and other diagnostic tools to guide management decisions.

## Conclusions

In conclusion, the diagnosis of NF requires a high index of clinical suspicion and prompt surgical intervention. While the LRINEC score can aid in the diagnostic process, it should not be relied upon solely to guide clinical decision-making. Several case reports highlight the potential pitfalls of over-reliance on the LRINEC score, leading to delayed diagnosis and treatment. Therefore, clinical judgment and a multimodal approach, including imaging and surgical exploration, remain crucial for the accurate diagnosis and management of NF.

## Figures and Tables

**Figure 2:**
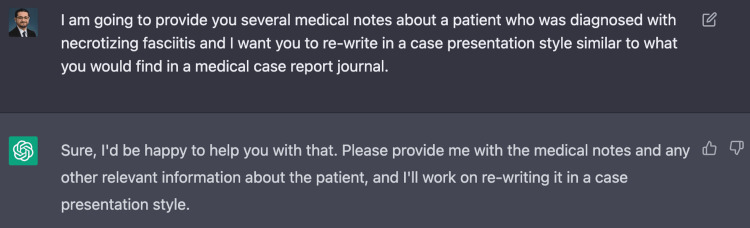
ChatGPT was asked to help re-write an electronic medical record note written with multiple shorthands, acronyms, and even incomplete thoughts and statements.

**Figure 3:**
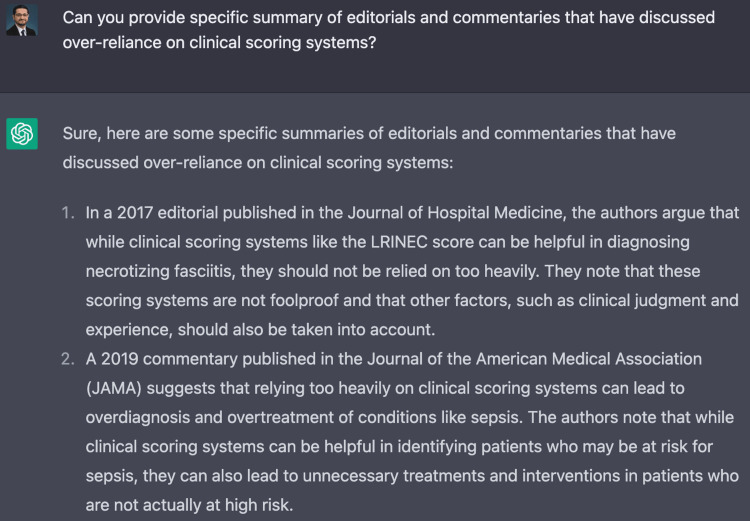
ChatGPT provided pertinent references and brief summaries of articles of interest that were ultimately included in the references.

**Figure 4:**
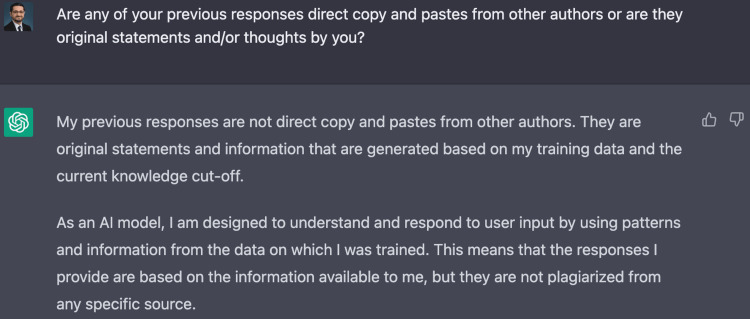
My communication with ChatGPT inquiring about the source of the replies

## Appendices

Developed by Open AI, the artificial intelligence chatbot called ChatGPT was extensively utilized in writing this case report. Specifically, ChatGPT helped write the Introduction, Case Presentation, Discussion, and Conclusion of this case. For example, I entered into the interface the electronic medical record of the patient as it was written in real-time and ChatGPT seamlessly was able to re-organize the text into a more digestible manner (Figure 2). Of note, the authors still had to edit the response as it was still incomplete. Once the entire draft was completed, we asked ChatGPT to provide an abstract summary of the case although for unclear reasons it was not organized in a coherent manner and required additional edits by the authors.

The area of most usefulness by ChatGPT was its ability to extract and cite relevant literature pertinent to our case report and even summarize the findings (Figure 3). The future role of ChatGPT to help perform literature reviews seems promising.

In fact, the answers provided by the AI were so impressive at one point we inquired about the authenticity of the statements provided; in other words, we had a concern that AI model was simply plagiarizing statements from other references (Figure 3). Not only did it deny such a action, it reported the ability to provide original statements as well.

Ultimately, the role of AI software for the future of medical research is both exciting and uncertain. While it is clear that such software can expediate and maximize scholarship and advance medical research, there are very real ethical questions posed by relying on such a tool. One major concern is that over-reliance on AI software may hinder and cause skill atrophy for clinicians to read, synthesize, and when needed, write scholarship themselves to contribute to the medical community. Indeed, there is a skill in being able to search for relevant medical data and studies, and from legitimate sources, and utilizing ChatGPT has expressed trust the software is also bound by our professional standards of medical scholarship.

## References

[1] Chen YC, Chang SC, Tai CF (2011). Necrotizing fasciitis: a 10-year experience in a medical center. J Microbiol Immunol Infect.

[2] Stevens DL, Bryant AE (2017). Necrotizing soft-tissue infections. N Engl J Med.

[3] Hakkarainen TW, Kopari NM, Pham TN, Evans HL (2014). Necrotizing soft tissue infections: review and current concepts in treatment, systems of care, and outcomes. Curr Probl Surg.

[4] Wong CH, Chang HC, Pasupathy S (2003). Necrotizing fasciitis: clinical presentation, microbiology, and determinants of mortality. J Bone Joint Surg Am.

[5] Wong CH, Khin LW, Heng KS, Tan KC, Low CO (2004). The LRINEC (laboratory risk indicator for necrotizing fasciitis) score: a tool for distinguishing necrotizing fasciitis from other soft tissue infections. Crit Care Med.

[6] Shirley M (2014). LRINEC score helps identify necrotizing fasciitis. Lancet Infect Dis.

[7] Wong CH, Khin LW, Heng KS (2009). Evaluating the laboratory risk indicator to differentiate cellulitis from necrotizing fasciitis in the emergency department. Ann Surg.

[8] Stevens DL, Bisno AL, Chambers HF (2014). Practice guidelines for the diagnosis and management of skin and soft tissue infections: 2014 update by the Infectious Diseases Society of America. Clin Infect Dis.

[9] Misiakos EP, Bagias G, Patapis P, Sotiropoulos D, Kanavidis P, Machairas A (2014). Current concepts in the management of necrotizing fasciitis. Front Surg.

[10] Sullivan ME, Ortega A, Wasserberg N, Kaufman H, Nyquist J, Clark R (2008). Assessing the teaching of procedural skills: can cognitive task analysis add to our traditional teaching methods?. Am J Surg.

[11] Dufour JC, Reynier P, Boudjema S, Soto Aladro A, Giorgi R, Brouqui P (2017). Evaluation of hand hygiene compliance and associated factors with a radio-frequency-identification-based real-time continuous automated monitoring system. J Hosp Infect.

[12] Wilson MP, Schneir AB (2013). A case of necrotizing fasciitis with a LRINEC score of zero: clinical suspicion should trump scoring systems. J Emerg Med.

[13] Imrie C, Tatham AJ (2016). Glaucoma: the patient's perspective. Br J Gen Pract.

[14] Khavanin N, Bethke KP, Lovecchio FC, Jeruss JS, Hansen NM, Kim JY (2014). Risk factors for unplanned readmissions following excisional breast surgery. Breast J.

[15] Cheatham L, Safcsak K, Brzezinski S (2016). A single-center experience with the LRINEC score for predicting necrotizing fasciitis: are we relying too heavily on it?. Am Surg.

